# Effectiveness of Interventions to Reduce Blood Pressure in Older Adults with Hypertension: A Network Meta-Analysis

**DOI:** 10.1093/geroni/igaf122.3214

**Published:** 2025-12-31

**Authors:** Noppawan Piaseu, Phatcharaphon Whaikid

**Affiliations:** Ramathibodi Hospital, Bangkok, Krung Thep, Thailand; Mahidol University, Bangkok, Krung Thep, Thailand

## Abstract

This study evaluated the effectiveness of interventions in managing blood pressure among older Thai adults. A systematic search was conducted in PubMed, Embase, Scopus, and the Thai-Journal Citation Index Centre for studies published from 2018 to 2024. Eligible studies included randomized controlled trials and quasi-experimental designs. Two independent reviewers assessed study quality using the Joanna Briggs Institute critical appraisal tool. A network meta-analysis was performed using Stata software. The study protocol was registered on PROSPERO (CRD42025634594). A total of 27 studies, including 1,828 participants over 60, met the inclusion criteria and were included in the analysis of three interventions: Self-Management (SM), Theory-Based Health Behavior Modification (TBHBM), and Exercise. The network meta-analysis revealed that Exercise was the most effective intervention for lowering systolic blood pressure (SBP). Compared to SM, Exercise significantly reduced SBP (MD = -6.75 mmHg, 95% CI: -18.59, -5.09), followed by TBHBM (MD = -0.80 mmHg, 95% CI: -11.54, -9.91). For diastolic blood pressure (DBP), Exercise was also the most effective intervention. Compared to SM, Exercise led to a greater reduction in DBP (MD = -4.98 mmHg, 95% CI: -12.80, 2.84), and compared to TBHBM, the reduction was also greater (MD = -3.14 mmHg, 95% CI: -10.10, 4.12). This provides evidence supporting health behavior modification as an effective strategy for blood pressure management in older Thai adults. Exercise was most effective for SBP reduction, while TBHBM had the most significant impact on DBP. These findings emphasize the importance of tailored interventions to optimize hypertension management in aging populations.

